# Multilayer Technique Using Calcium Hydroxylapatite Biostimulation With Different Dilutions in the Lateral Face

**DOI:** 10.1093/asjof/ojae049

**Published:** 2024-12-12

**Authors:** Claudia Hernandez, Bianca Viscomi, Gladstone Faria, Rossana Vasconcelos, Carolina Schneider, Jorge Moreno, Mariana Muniz

## Abstract

**Background:**

There seems to be an interdependency of superficial structures on deeper layers, so that aging-related changes in 1 layer may lead to changes to the adjacent layers. Following the same rationale, treatment of 1 area may influence other neighboring aesthetic units. A more holistic approach would encompass soft-tissue repositioning and regenerative biostimulation, aiming for improvement of skin quality by increasing skin's collagen content.

**Objectives:**

To describe the use of calcium hydroxylapatite (CaHA) in different presentations for soft-tissue repositioning and improvement of skin quality in the same session.

**Methods:**

Males or females between 40 and 60 years of age, with normal BMI, mild facial laxity, underwent supraperiosteal injection of undiluted CaHA for focal biostimulation along the zygomatic arch, in the mandible angle and in the prejowl area, followed by treatment of diluted CaHA in the posterior temporal area, and the remainder in the premasseteric area in the same session, with follow-up pf at least 90 days. Investigator assessment was evaluated using the Global Aesthetic Improvement Scale.

**Results:**

Out of 6 treated patients (median age of 44.5 years), 66% were deemed as improved (Grade 3) for the treatment of upper third of the face, whereas 83% of the patients were assessed as having at least improved for the mid and lower thirds of the face. Only mild adverse events were reported.

**Conclusions:**

The technique described in this pilot study provides a full-face approach with CaHA based on the current concepts of the line of ligaments and facial biomechanics. Further studies are needed to validate the results.

**Level of Evidence: 4:**

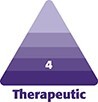

The aging process may be split into 2 major components, with the degree of overlap depending on each individual.^1^ The intrinsic aging is mostly associated with chronological age, while extrinsic aging, stems from environmental exposure to a myriad of factors, including sun, pollutants, and diet.^1^ A broader concept “exposome” was suggested as the cumulative measure of environmental influences and the associated biological response throughout the lifespan of a person,^2,3^ being responsible for as much as 80% of the aging process.^4^

Facial aging occurs simultaneous and progressively in all facial structures (from skin, adipose tissues, superficial musculoaponeurotic system, muscles, and bones) with the onset and the speed of age-related changes differing for each structure and individual.^5^ Bone suffers remodeling in certain areas, such as expansion of the supraorbital ridges, increase in the pyriform aperture, resorption in the anterior and inferior aspects of the maxilla, and giving the appearance of a retrusion of the face.^6,7^ Some superficial fat pads, such as the nasolabial superficial fat pad, tend to suffer repositioning or hypertrophy.^5,8^ The facial muscle tone may increase (eg, procerus) or decrease clinically being translated as accentuation of skin creases and skin wrinkling.^5^ Changes to the bone and other structures, where ligaments are inserted, lead to altered mechanical function so that a ligament may fatigue, leading to the appearance of sagging of the respective fat compartment, as seen in the mechanism of jowling, which also includes the pull down by the platysma muscle.^5,9^ Collagen and elastin content of the skin suffer reduction as a result of decrease in synthesis and excessive breakdown of the extracellular matrix by upregulated matrix metalloproteinases,^10^ clinically translated as sagging and wrinkling.

Although each anatomical layer of the face undergoes an aging process of its own, there is an interdependency of superficial structures on deeper layers, so that a change in 1 layer may lead to changes to the adjacent layers.^8^ Following the same rationale, interdependency is also observed during treatment in 1 area, which may influence other neighboring aesthetic units,^9,11^ supporting the concept that for rejuvenation, the face should be approached as a single unit. Previous studies have provided objective evidence that a local volumizing effect achieved with soft tissue in the lateral face can translate into a repositioning effect.^9,12-16^ For instance, temporal injections lateral to the “line of ligaments,” a single line formed by true osteocutaneous ligaments, extending from the temporal crest to the mandible,^17^ lead to a repositioning effect of the mid and lower face,^9,12-16^ because the fascial continuity of the lateral face can transmit treatment effects beyond the local boundaries of the temporal region.^12^ Although, in most reported approaches in the literature, the main objective was soft-tissue repositioning, we suggest a more holistic approach that would also encompass regenerative biostimulation targeting an improvement in skin quality by repairing the extracellular matrix.^18,19^ Albeit hyaluronic acid (HA) may induce neocollagenesis, calcium hydroxylapatite (CaHA) resulted in more active, physiologic remodeling of the extracellular matrix compared with HA, by stimulating a 2-step process whereby collagen Type I gradually replaced collagen Type III.^20^ Immunohistochemical data of increase in collagen Type I synthesis correlated with improvements in skin elasticity and pliability, as evaluated by cutometry and increase in dermal thickness, as assessed by ultrasound.^21^ Thus, in this context, we sought to describe herein 6 clinical cases, illustrating the technique, a holistic approach aiming for facial rejuvenation combining soft-tissue structural repositioning and improvement of skin quality with CaHA.

## METHODS

All procedures performed in this report involving human patients were in accordance with the ethical standards of the institutional and/or national research committee and with the 1964 Declaration of Helsinki and its later amendments or comparable ethical standards.

### Ethics Approval

The case report was approved by a centralized institutional review board (68346223.0.0000.0081 and 65837122.9.1001.8125). Written informed consent has been provided by all the patients, by which they agreed to the use and analysis of their data.

We report 6 cases of facial rejuvenation combining structural repositioning and global biostimulation techniques with the use of undiluted CaHA with 0.3% powdered lidocaine hydrochloride (CaHA(+); Radiesse Plus; Merz Pharmaceuticals GmbH, Frankfurt, Germany) and diluted CaHA (Radiesse; Merz Pharmaceuticals GmbH) between September 2021 and March 2022. Eligible patients were males or females, between 40 and 60 years of age, with normal BMI (18.5-24.9), mild facial laxity, who sought facial rejuvenation and agreed to participate in the study. Exclusion criteria included previous facial surgery, use of fillers or biostimulators within the last 12 months, the presence of acne scars, and/or use of hormonal therapy. Photographs were taken at baseline, immediately after injection and after a follow-up interval of at least 90 days after the procedure, up to 9 months after treatment. Two-dimensional photographs were taken with a digital camera (Vectra Software, Canfield, NJ). Standardized indirect light, distance of the camera, as well as aperture and speed were controlled. Pre and post images were compared with the VECTRA 3D system (Canfield Scientific, Fairfield, NJ) for clinical and volumetric evaluation by referencing the preinjected image to the postinjection images in each patient.

Clinical efficacy was evaluated by patients and blinded investigators at 3 months using the Global Aesthetic Improvement Scale (GAIS) for each of the facial thirds (ie, upper, mid, and lower thirds). The 5-point scale ranging from 1 (very much improved) to 5 (worse) rates global aesthetic improvement in appearance compared with pretreatment levels.

### Technique

**Figure 1.**
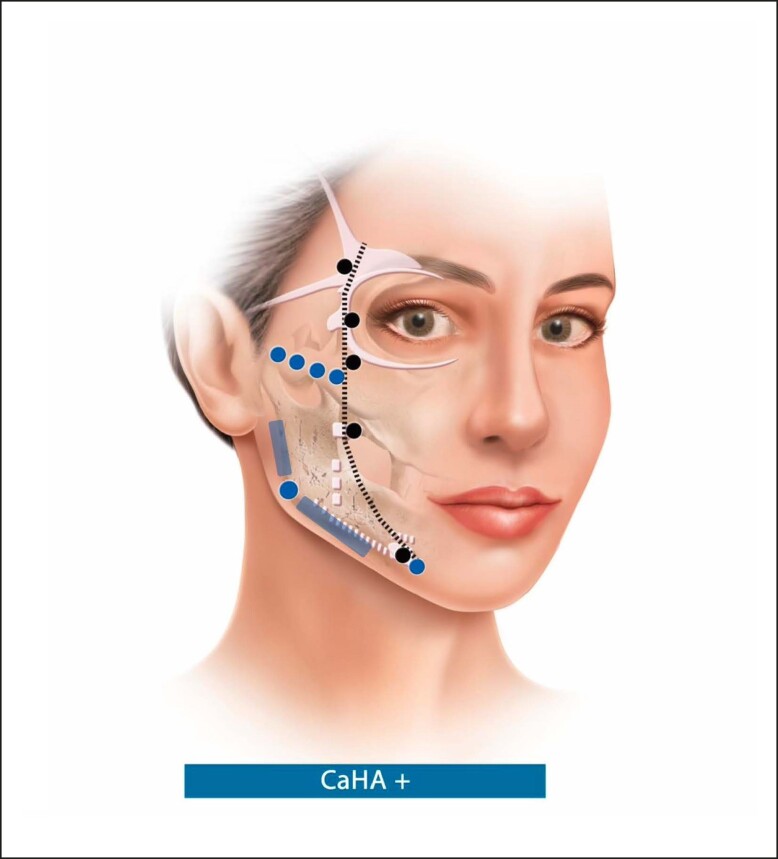
Step 1: focal biostimulation: black dots and black dotted line refer to the line of ligaments. White structures: ligaments, adhesions, and septums of the face. Blue dots: Supraperiosteal boluses of CaHA(+) for Restoration/support of the bony landmarks of the lateral face. In case more volume should be needed, increase the number of boluses and inject less volume per bolus to achieve a more natural result. If further mandibular definition is needed, subdermal injections of CaHA(+) along the mandible line and ramus (blue line). CaHA, calcium hydroxylapatite.

The technique can be used for any gender, with the most suitable patient having mild-to-moderate skin laxity and normal BMI. This technique takes into account the line of ligaments (ie, lateral orbital thickening, temporal ligamentous adhesion, zygomatic ligament, and mandibular ligament aligned in 1 line), in order to delimit the lateral and medial aspects of the face^13^ and it is paramount that all injections are performed lateral to the line of ligaments (Figure 1).

**Figure 2.**
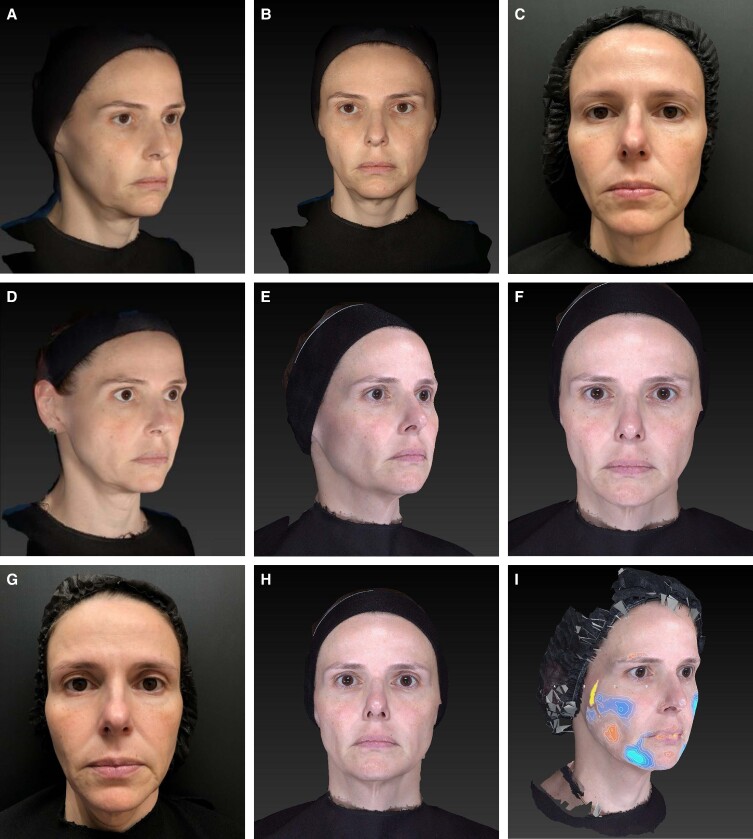
Photographs of a 42-year-old female. Treatment schema: Step 1: 3 boluses (0.15/0.15/0.1 mL) along zygomatic arch; 0.2 mL in the mandible angle and 0.1 mL bolus in the prejowl area (1.5 mL of CaHa(+); 0.75 mL/side); Step 2: 0.5 mL in the anterior temporal area, 1 mL in the posterior temporal area, and 1.5 in the premasseteric area (3 mL per side; 6 mL of diluted CaHA per session; 2 × 1.5 mL syringes). Images taken with Vectra Software at baseline (A, B, and C), after 90 days (D), 6 months (E, F, and G), and 9 months (H) after the treatment session. Volumetric evaluation with Vectra Software (I; 6 months) illustrates changes in volume, with blue areas denoting increased projection, whereas red–orange–yellow areas being related to decreased projection. GAIS evaluation at 90 days: blinded Evaluator 1: upper third 3 (improved), mid-third 2 (much improved), lower third 2 (much improved); blinded Evaluator 2: upper third 4 (no change), mid-third 2 (much improved), lower third 2 (much improved). CaHA, calcium hydroxylapatite; GAIS, Global Aesthetic Improvement Scale.

**Figure 3.**
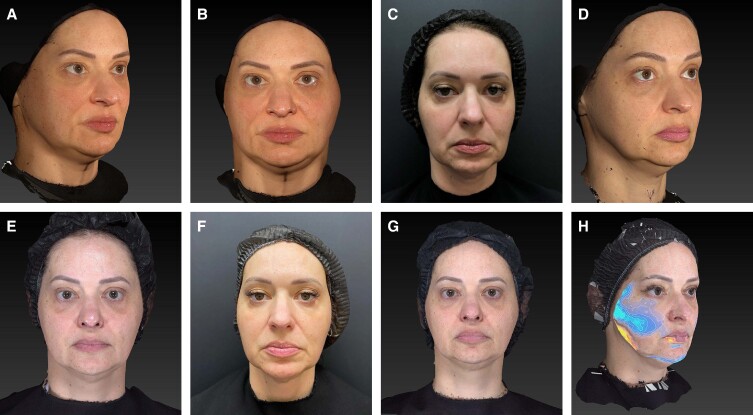
Clinical Case 5: a 45-year-old female. Treatment schema: Step 1: 3 boluses (0.15/0.15/0.1 mL) along zygomatic arch; 0.2 mL in the mandible angle and 0.1 mL bolus in the prejowl area (1.5 mL of CaHa(+); 0.75 mL/side); Step 2: 0.5 mL in the anterior temporal area, 1 mL in the posterior temporal area, and 1.5 in the premasseteric area (3 mL per side; 6 mL of diluted CaHA per session; 2 × 1.5 mL syringes). Images taken with Vectra Software at baseline (A, B, and C), 90 days (D), 6 months (E and F), and 9 months (G) after the treatment session. Lower face soft-tissue repositioning is better observed by volumetric evaluation with Vectra Software (H; 6 months), which illustrates changes in volume, with blue areas denoting increased projection, whereas red–orange–yellow areas being related to decreased projection. GAIS evaluation at 90 days: blinded Evaluator 1: upper third 2 (much improved), mid-third 3 (improved), lower third 3 (improved); blinded Evaluator 2: upper third 3 (improved), mid-third 4 (no change), lower third 3 (improved). CaHA, calcium hydroxylapatite; GAIS, Global Aesthetic Improvement Scale.

The first step should be the placement of supraperiosteal boluses of CaHA(+) for focal biostimulation with a 27 G needle, in 3 points along the zygomatic arch (0.15/0.15/0.1 mL per injection point; total volume of 0.45 mL; Figure 1). In case more volume is needed, it would be advisable to increase the number of boluses to 4 and inject less volume per bolus to achieve a more natural result. Moreover, one 0.2 mL bolus of CaHA(+) should be injected in the mandible angle and one 0.1 mL bolus in the prejowl area, totaling 0.75 mL/side (Figures 2, 3). Usually, 1 to 2 syringes of CaHA(+) may be needed in 1 session. This step aims for the restoration/support of the bony landmarks of the lateral face. Although we suggest the use of a 27 G needle, the result can be achieved with cannula if the injector feels more comfortable with the use of this device. Further definition of the mandibular line can be achieved through subdermal injections of CaHA(+) along the mandible line and ramus.^22^

**Figure 4.**
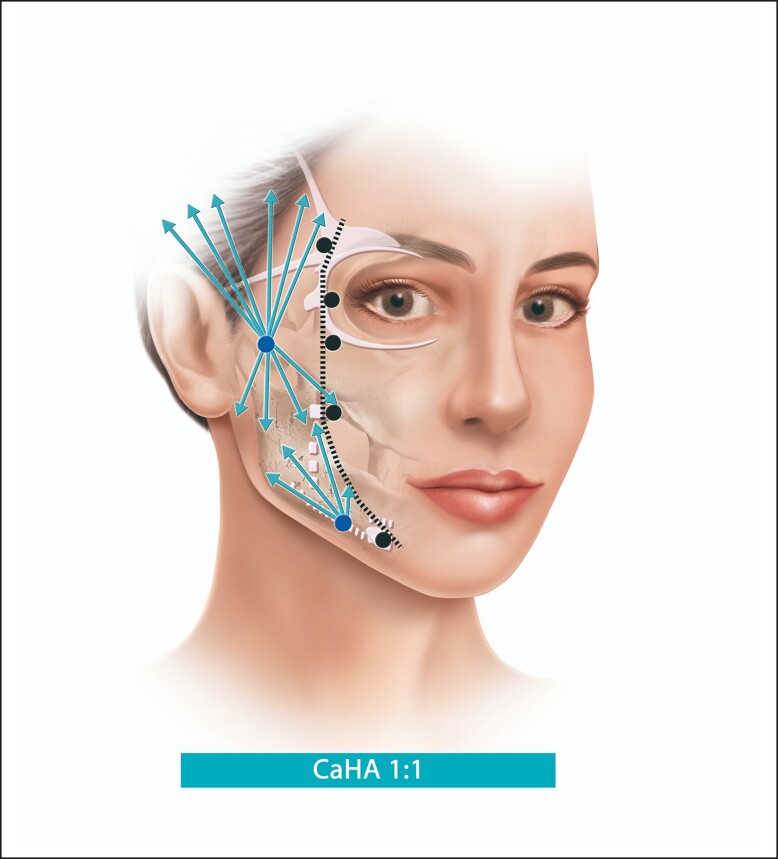
Step 2: global biostimulation: through an entry point in the transition of the body and the frontal process of the zygomatic bone area (blue circle), diluted CaHa (1:1 dilution) should be injected in the subdermal plane in the anterior temporal area, in the posterior temporal area, and the remainder in the premasseteric area. If needed for premasseteric approach, a second entry point in the jowl may be used. CaHA, calcium hydroxylapatite.

After the deep injections have been performed, an entry point in the transition of the body and the frontal process of the zygomatic bone allows safe access to both the temple and the premasseteric area.^23^ If needed for premasseteric approach, a second entry point in the jowl may be used. CaHA diluted with a solution of 0.5 mL lidocaine without epinephrine and 1 mL of saline (1:1 dilution) should be injected with a 22 to 25 G cannula in 0.1 mL vectors in the subdermal plane (Figure 4). The suggested volume per area and per side is 0.5 mL in the anterior temporal area, 0.5 to 1 mL in the posterior temporal area, and the remainder in the premasseteric area, totaling 3 mL of diluted CaHA per side (6 mL of diluted CaHA per session; 2 × 1.5 mL syringes or 1 syringe × 3 mL; Figures 2, 3). A massage to better accommodate the product in the injected areas should be performed. All patients were treated only once. Nonetheless, the treatment can be repeated according to the patient's needs, respecting the 90-day interval (Video).

## RESULTS

### Demography

Patients enrolled were females, between 40 and 60 years of age (median age 44.5 years) and no relevant medical history (eg, use of medications that cause bleeding, previous facial surgery, allergies, urticaria, angioedema; presence of active infections or other inflammatory processes in the area to be treated, dental treatment, use of permanent fillers, any injectable procedure or facial surgery within the last 18 months). The median follow-up of the patients was 7.5 months.

### Global Aesthetic Improvement Scale Evaluation

Two-thirds of the patients were deemed as improved (Grade 3) for the treatment of upper third of the face, whereas 1 patient did not present any change. For 1 patient, there was discordance between the assessment of the 2 blinded investigators if improvement or no change was observed. For the mid (4/6 improved; 1/6 much improved) and lower thirds of the face, 83% of the patients were assessed as having at least improved (2/6 improved; 3/6 much improved). One patient did not present any improvement of the upper or lower thirds but was assessed as having improved in the mid-third of the face.

### Adverse Events

Adverse events reported were mild in intensity and transient, being mostly ecchymosis.

## DISCUSSION

For a more holistic approach to facial aging, the technique comprises global biostimulation for skin quality improvement and focal biostimulation with the aim to induce a nonsurgical soft-tissue repositioning effect. The superficial fascial system is not only a single, flat layer of connective tissue but a 3-dimensional composite of fat and fibrous collagenous tissue, comprising the dermis, superficial fat, subdermal septae, and superficial fascia, that serves to transmit the movement of the underlying facial muscles to the overlying skin.^16^ Tightening of the superficial fascial system has virtually become an integral part of face-lift surgery as it is currently performed^24^ and would be the ideal target for both the global and focal biostimulation.

Besides being a surface landmark, the line of ligaments is also an anatomical and functional boundary between the medial and lateral face.^17^ The layers of the face are organized differently medial or lateral to the line of ligaments: In the medial face, the fascia, the muscles, and the encapsulated superficial and deep fat compartments that fill the spaces between the fascial layers are arranged in an oblique manner. Moreover, when the facial muscles change their plane, they connect these layers with each other.^13^ On the other hand, in the lateral face, the fascial layers are arranged in a parallel disposition, without the presence of muscles of facial expression or deep facial fat compartments.^13,25^

In terms of functionality, the muscles of mastication are exclusively located in the lateral face, whereas the muscles of facial expression are predominately located in the medial face.^25,26^ The line of ligaments is the most lateral aspect where the muscles of facial expression originate from the bone,^13^ being plausible to expect more active movement in the medial face.^27^ Nevertheless, passive mobility (ie, greater facial skin displacement during postural change) is greater in the lateral face as there is no strong connection of the skin and superficial fat compartments to the underlying bone or deep fascia, owing to the absence of major facial ligaments and muscles attached to the overlying skin. This renders the soft tissues in the lateral face more prone to gravity and age-related descent.^25,27^

Approaches that respect this anatomical and biomechanical differences of the medial and lateral aspects of the face have been shown to have better outcomes,^12-14^ since injections performed medial and lateral to the line of ligaments render different outcomes. Lateral injections have a soft-tissue repositioning effect, while medial injections a more volumizing effect.^13,17^ In the technique described herein the injections are performed in alignment with the facial biomechanics principle that the lateral face should be approached first.^25^ The soft-tissue repositioning effect obtained by injecting the lateral face first reduces the volume of filler needed to achieve the desired outcome when compared with injecting the medial face first.^13^ Furthermore, when aiming for a soft-tissue repositioning effect, besides starting with the lateral aspect of the face, the sequence of areas to be injected should also be taken into account. A study applying 3-dimensional surface volumetric and skin vector displacement analysis observed that soft-tissue injectables can induce not only local changes, but also local and regional soft-tissue repositioning effects, depending on the region of injection.^16^ CaHA is well suited for injection in the supraperiosteal plane since in areas with nondistensible tissue, the lifting potential of the filler must be high to provide sufficient projection.^28,29^ The high viscosity and high elastic modulus (*G*′) of CaHA ensures that it remains where injected and withstand gravitational forces, efficiently providing composite lift and support to the overlying tissues.^28^ CaHa injection in the malar location induced a noticeable lifting effect, with recruitment of ptotic tissue and lateral movement of the Nasolabial fold/crease,^30^ as observed in Figure 2. Therefore, the first approach in the technique should be the placement of boluses along the zygomatic arch, for focal neocollagenesis and soft-tissue repositioning purposes. Even though the lower third was not injected, the observed improvement as per GAIS assessment was greater in the lower third. This could be explained by the presence of short subdermal fibrous septae that separate the temporal and preauricular compartments, mainly along the zygomatic arch,^31^ which work as a lever arm to lift and tighten inferiorly located facial tissues. Moreover, the deep supraperiosteal plane being addressed first allows recreation of the bony support for the overlying facial soft tissues to reposition and move with facial expressions more naturally, without interference of the soft-tissue filler, whereas targeting the superficial fat compartments first can result in a less natural facial appearance with movement.^14^ Also, the supraperiosteal plane is safer since the arterial vasculature usually travels in more superficial planes.^25,29^

The second step of this technique would be global biostimulation, with subdermal injections of CaHA. In the temple area, studies have previously shown that deep supraperiosteal injections alone have limited soft-tissue repositioning effects, while the combined effects of subdermal injections of the temple, lateral midface, and mandibular angle can induce repositioning of the total lateral face,^15,16^ improving the appearance of the jawline, jowls, labiomandibular sulcus and even in some cases the nasolabial sulcus.^32^ Architectural changes induced by product deposition in the subdermal fatty layer modify the biomechanics of the superficial fascial system (ie, tension within that functional unit), clinically translated into the desired soft-tissue repositioning effect.^16^ Even though the sample size of this pilot study was small, 83% of the patients were assessed as having at least improved in GAIS evaluation for the mid and lower thirds (Figure 3), corroborating previous findings that injections in the posterior temple result in regional soft-tissue repositioning effects, including the middle and lower face.^32^

Global biostimulation also targets skin quality improvement. Fibroblastic dysfunction, in terms of abnormal proliferation, migration, differentiation, and synthesis of collagen, is one of the main markers of skin aging and structural and functional skin changes.^33^ A significant decrease in collagen Type I production and impairment of migratory capacities were observed in wrinkle fibroblasts compared with normal fibroblasts. Addition of CaHA was able to restore the contractile properties of wrinkle fibroblasts to the same level as normal fibroblasts, displaying positive effects on the aging process.^33^ Moreover, CaHA displays a dual mechanism of action, clinically translated into immediate and long-lasting results. After an initial immediate volume replacement, the carrier gel is gradually absorbed over time, leaving the CaHA microspheres to induce neocollagenesis,^34^ proteoglycans and elastin increase,^35^ indicating it can induce remodeling of all aspects of the extracellular matrix.^19^ The neocollagenesis is progressive, in which collagen Type III is gradually replaced by collagen Type I, with significant peak collagen Type III expression at 4 months (*P* < .00001), and significant collagen Type I expression at 4 and 7 months postinjection (*P* = .04 and *P* < .00001 respectively), entailing sustained long-term correction.^21,36^ Elastin expression is supposed to follow a similar pattern to that of collagen Type I, as well as angiogenesis, suggesting that CaHA improved blood flow and therefore nutrient supply to the injected area of the skin. Yutskovskaya and Kogan demonstrated that dermal remodeling was associated with an increase in dermal thickness, and improved skin mechanical properties, including elasticity and pliability.^21^ Improvement in dermal thickness is expected to have clinical significance from D120 onwards,^37^ and even though skin biopsy was not performed in the current study, the GAIS score improvement was observed in the current study from D90 onwards (Figure 2), but even higher at D180 evaluation.

In a systematic review of adverse reactions to the injection of face and neck aesthetic filling materials, silicone was the most reported product (19.7%), followed by HA (15.5%), with CaHA responsible for 5.6% of the reported cases.^38^ CaHA can be associated with local, short-term injection-related adverse events, which are usually deemed as mild and resolve within a few days,^39^ infection,^40^ and long-term adverse events (eg, nodules). A literature search with >5081 treatments with CaHA in 2779 patients reported 3% of AEs, out of which most consisted of nodules (*n* = 166; 96%) and persistent inflammation/swelling (*n* = 2; 2%), persistent erythema (*n* = 1; 1%), and overcorrection (*n* = 1; 1%).^41^ Of the reported nodules, 49% occurred in dynamic areas (prone to nodule formation) such as lips and perioral areas, and most cases did not require treatment.^41^ In the current study, most adverse events observed were of mild intensity, mostly related to ecchymosis.

It is important to highlight that there is no one-size-fits-all. All volumes and injection points described herein provide a basis upon which the treatment should be tailored to the patient's needs. Some patients may need to repeat the session, respecting the 120-day interval for reevaluation of CaHA treatment^21^ or require association of other therapies (eg, botulinum toxin and lasers). Patients with jowl formation, for instance, may require treating the mandibular angle in the postjowl area, not just in the prejowl sulcus, so that the descended fat pads are fully camouflaged. In addition, it is important to point out that although biostimulation with CaHA has been reported with positive results,^20,21,34,35,42-44^ consensus on objective and standardized parameters is still lacking.

This preliminary study's limitations are the small sample size not powered for statistical analysis, lack of skin biopsy and objective evaluation of CaHA biostimulation, so that larger studies are needed to validate the results. Nevertheless, it highlighted the importance of targeting the area lateral to the line of ligaments, to promote a lifting effect without the need of large amounts of HA, which can lead to unnatural results.

## CONCLUSIONS

The technique described herein provides a full-face approach based on the current concepts of the line of ligaments and facial biomechanics. The unique characteristics of CaHA (high elasticity and viscosity), as well as its ability to induce long-term collagen formation^21,36^ and promote improvement of the extracellular matrix^35^ may render great versatility, appropriate for focal biostimulation with the aim to induce a nonsurgical repositioning of soft tissues and global biostimulation with the objective of improvement of skin extracellular matrix and skin quality.
